# ENGAGING OLDER ADULTS AND CAREGIVERS AS STAKEHOLDERS IN THE ARTIFICIAL INTELLIGENCE AND TECHNOLOGY COLLABORATORY

**DOI:** 10.1093/geroni/igad104.2334

**Published:** 2023-12-21

**Authors:** Paavani Jain, Nancy Schoenborn, Thomas Cudjoe

**Affiliations:** Johns Hopkins University School of Medicine, Baltimore, Maryland, United States; Johns Hopkins University School of Medicine, Baltimore, Maryland, United States; Johns Hopkins University School of Medicine, Baltimore, Maryland, United States

## Abstract

Artificial Intelligence (AI) holds the unique potential to capture older adult functional and behavioral diversity and improve geriatric care. Yet a gap exists between new technologies and the real-world needs of their target populations. Currently in the United States, three Collaboratories support novel AI projects in Aging Research. As the Stakeholder Engagement Core of the Johns Hopkins AI Collaboratory, in this poster, we share early lessons of forming a ‘Council’ of stakeholders – a group with the charge of informing pilot AI projects longitudinally. The Council was envisioned to include 10-15 persons: older adults, caregivers, and clinicians. We adopted different outreach techniques for clinicians versus non-clinicians; leveraged existing relationships with affiliated Memory and Social Clinics; individually approached community dwelling older adults and caregivers including homebound older adults and older adults with disabilities; and reached out to clinicians and advocates known to be committed to geriatric care. We developed plain language materials and explained the meaning of ’AI’ and ’Stakeholder Engagement’. Five out of 5 outreached clinicians, and 10 out of 20 older adults/caregivers agreed to serve on the Council. Many older adults on our Council had difficulty using their email and computer and required support. Several caregivers declined due to high caregiving demands and many older adults with multiple chronic conditions declined due to high personal needs; all reiterated their interest in the topic area despite declining participation. Future novel-technology projects should ensure inclusion of diverse stakeholders; incorporating a flexible plan of engaging high-needs older adults and caregivers is required.

